# 

**Published:** 1874-01

**Authors:** 


JANUARY, 1874.

OFFICE.
LIBRARY.

### THE AMERICAN JOURNAL OF DENTAL SCIENCE.

EDITED BY

F. J. S. GORGAS, M. D., D. D. S.

VOL. 7.—THIRD SERIES.—No. 9.

BALTIMORE:

SNOWDEN & COWMAN.

LONDON:

TRUBNER & CO., 60 PATERNOSTER ROW.

1874.

$2.50 PER ANNUM.

PAYABLE IN ADVANCE.

